# The District Committees

**Published:** 1888-07

**Authors:** K. P. Moore

**Affiliations:** Secretary


THE DISTRICT COMMITTEES, heretofore appointed by the Nominating Committee, at the sitting of the Georgia Medical Association, will hereafter be appointed by a Special Committee composed of one from each Congressional District. This committee will hereafter be appointed by the Secretary. This duty has been performed, as we learn from the following communication kindly addressed to the managing editor, by our worthy Secretary:

COMMITTEE OF DISTRICTS.

*Dr. R. C. Word, Atlanta, Ga,:*

At the late meeting of the Georgia Medical Association the following resolution was passed:

"*Be it resolved*, That the power of the Nominating Committee be restricted to the nomination of officers of the Association, and that a Special Committee consisting of one member from each Congressional District shall be appointed by the Secretary, whose duty shall be to name the members in each district to report upon the special subjects for each succeeding meeting, and that the committee be known as the Committee of Districts."

In accordance with the above resolution, I have appointed the following names as that committee, and at your request send them to you for publication in the SOUTHERN MEDICAL RECORD: First District, R. J. Nunn, Savannah; Second District, R. B. Doster, Blakely; Third District, A. A. Smith, Hawkinsville; Fourth District, A. W. Griggs, West Point; Fifth District, G. G. Roy, Atlanta; Sixth District, R. O. Cotter, Macon; Seventh District, T. M. Holmes, Rome; Eighth District, G. W. Mulligan, Washington; Ninth District, L. G. Hardman, Harmony Grove; Tenth District, Eugene Foster, Augusta.

Respectfully,

K. P. MOORE, *Secretary.*

